# Musically Cued Gait-Training Improves Both Perceptual and Motor Timing in Parkinson’s Disease

**DOI:** 10.3389/fnhum.2014.00494

**Published:** 2014-07-07

**Authors:** Charles-Etienne Benoit, Simone Dalla Bella, Nicolas Farrugia, Hellmuth Obrig, Stefan Mainka, Sonja A. Kotz

**Affiliations:** ^1^Department of Cognitive Psychology, University of Finance and Management in Warsaw, Warsaw, Poland; ^2^Max Planck Institute for Human Cognitive and Brain Sciences, Leipzig, Germany; ^3^Movement to Health Laboratory (M2H), EuroMov, Montpellier-1 University, Montpellier, France; ^4^Institut Universitaire de France, Paris, France; ^5^Goldsmiths, University of London, London, UK; ^6^Clinic for Cognitive Neurology, University Hospital, University of Leipzig, Leipzig, Germany; ^7^Neurologisches Fachkrankenhaus für Bewegungsstörungen/Parkinson, Beelitz-Heilstätten, Germany; ^8^School of Psychological Sciences, The University of Manchester, Manchester, UK

**Keywords:** Parkinson disease, auditory cueing, timing, motor behavior, perception

## Abstract

It is well established that auditory cueing improves gait in patients with idiopathic Parkinson’s disease (IPD). Disease-related reductions in speed and step length can be improved by providing rhythmical auditory cues via a metronome or music. However, effects on cognitive aspects of motor control have yet to be thoroughly investigated. If synchronization of movement to an auditory cue relies on a supramodal timing system involved in perceptual, motor, and sensorimotor integration, auditory cueing can be expected to affect both motor and perceptual timing. Here, we tested this hypothesis by assessing perceptual and motor timing in 15 IPD patients before and after a 4-week music training program with rhythmic auditory cueing. Long-term effects were assessed 1 month after the end of the training. Perceptual and motor timing was evaluated with a battery for the assessment of auditory sensorimotor and timing abilities and compared to that of age-, gender-, and education-matched healthy controls. Prior to training, IPD patients exhibited impaired perceptual and motor timing. Training improved patients’ performance in tasks requiring synchronization with isochronous sequences, and enhanced their ability to adapt to durational changes in a sequence in hand tapping tasks. Benefits of cueing extended to time perception (duration discrimination and detection of misaligned beats in musical excerpts). The current results demonstrate that auditory cueing leads to benefits beyond gait and support the idea that coupling gait to rhythmic auditory cues in IPD patients relies on a neuronal network engaged in both perceptual and motor timing.

## Introduction

Idiopathic Parkinson’s disease (IPD) is one of the most common movement disorders. Although substantial progress has been made regarding the treatment of its cardinal motor symptoms, progressive brady- or akinesia, rigor, and tremor lead to disability and are a major challenge for the health care system (Elbaz et al., 2002). Clinically, gait disorder and postural instability leading to falls and fractures represent a major challenge for the patients as the disease progresses (Bloem, 1992; Koller and Montgomery, 1997; Grabli et al., 2012). However, even if motor deficits can be alleviated by a number of therapeutic regimes (Samii et al., 2004), cognitive and affective deficits emerge as additional challenges in the disease’s progression. These may dramatically influence patients’ quality of life and have been increasingly recognized to undermine independent living (e.g., Morris et al., 2001; Bloem et al., 2004).

While many motor symptoms in IPD can be alleviated by pharmacological treatment and deep-brain stimulation, effects on gait dysfunctions are rather meager, inconstant, and decrease over time (Blin et al., 1990; Grabli et al., 2012; Sharma et al., 2012). Therefore, physical therapy is an essential ingredient of IPD management. It is non-invasive, cost-efficient, and may slow the progress of the disease (Kwakkel et al., 2007). One way of compensating for gait disorders is the use of temporally predictable external cues (Lim et al., 2005; Spaulding et al., 2012). Rhythmic auditory cues have been shown to enhance gait spatio-temporal parameters such as speed and stride length (Lim et al., 2005). Typically patients are instructed to match their walking speed to a repeated isochronous sound (i.e., metronome) or to the beat of music (Thaut et al., 1996; McIntosh et al., 1997; Nieuwboer et al., 2007; de Bruin et al., 2010). Auditory cueing is efficient during stimulation (Howe et al., 2003; Willems et al., 2006; Arias and Cudeiro, 2008), but has also been shown to carry over to uncued gait after training (Nieuwboer et al., 2007). Some studies report a reduction of its benefit between 4 and 6 weeks after training (Thaut et al., 2001) with considerable deterioration almost to pre-test values after 12 weeks post-intervention (Nieuwboer et al., 2001). Other studies reported stable cueing benefits even after 4–6 weeks (Marchese et al., 2000; Lehman et al., 2005).

The neuronal mechanism underlying the sustained benefits of cueing-based training is largely elusive. It has been suggested that coupling movement to an external rhythmic stimulus reinforces compensatory neuronal networks enhancing motor behavior in IPD (Nombela et al., 2013; see also Kotz and Schwartze, 2011). One candidate is the sensorimotor network underlying temporal processing. Structuring actions in time is a key element to achieve precise and stable coordinated movement such as gait. As IPD patients also display timing deficits (Hallett, 2008; Allman and Meck, 2012; Wu et al., 2012), one cause of gait dysfunctions in self-initiated and self-paced movements may be reduced by enhancing general timing functions. Indeed, dopamine depletion, a characteristic of this disorder, leads to malfunctioning of the basal-ganglia–cortical circuitry crucially involved in timing (Wing, 2002; Coull et al., 2011; Merchant et al., 2013). An external rhythmic cue may modulate the activity within the impaired timing system. Presenting an external temporally predictable cue, to which patients can synchronize their steps, provides a regularizing temporal input to the timing system.

External cues generate temporal expectations (e.g., via a process called “entrainment”; Jones, 1976; Large and Jones, 1999; Large, 2008) allowing to predict when a next event (e.g., a step) should occur. This may facilitate movement optimization and execution. Rhythm-driven expectations can regularize and stabilize movement by synchronizing the timing of an action execution to the beat structure of an auditory stimulus (e.g., Nombela et al., 2013). Since this process is probably supported by a neural circuitry less affected by IPD, it may compensate progressive malfunctioning of the basal-ganglia–cortical circuitry (e.g., Lewis et al., 2007). The compensatory system is likely to involve cerebello-thalamo-cortical circuitry (see also Kotz et al., 2009; Kotz and Schwartze, 2011; Nombela et al., 2013), a hypothesis which has received some support from studies with IPD patients. Cerebellar connections to the SMA are hyperactivated when action is externally cued (Sen et al., 2010). Moreover, activity of the cerebellar anterior lobule is enhanced following 1-month of cueing-based training (del Olmo et al., 2006).

The aforementioned circuitry is likely to support auditory cueing and its effects on gait kinematics, and is part of a domain-general system affording both perceptual and motor timing (Coull et al., 2011; Merchant et al., 2013; Schwartze and Kotz, 2013). Therefore, it is expected that auditory cueing may not merely improve motor control during gait, but more generally to enhance performance in tasks involving perceptual and motor timing (e.g., hand tapping or duration discrimination). Effects of auditory cueing beyond gait kinematics have not been systematically investigated so far. The goal of the present study was therefore to test whether a 1-month training of gait via auditory cueing enhances perceptual and motor timing. This hypothesis was tested by submitting a group of IPD patients to the Battery for the Assessment of Auditory Sensorimotor and Timing Abilities (BAASTA), a comprehensive set of tasks for the assessment of perceptual and motor timing abilities. Patients were tested before, right after, and 1 month following the training. Impairment prior to training was assessed in comparison to a group of healthy age-matched participants (controls) considered as baseline. Improvement in perceptual and motor timing was expected in response to external cueing. Moreover, as long-term benefits of auditory cueing on gait have been observed several weeks after training (Nieuwboer et al., 2007), we addressed the question whether improvement in timing abilities would similarly persist 1 month after the training.

## Materials and Methods

### Participants

Fifteen right-handed non-demented patients (10 males) with IPD, aged 49–80 years (*M* = 67.2, SD = 7.5) participated in the study (see Table 1). Scores of the Unified Parkinson’s Disease Rating Scale (UPDRS) and staging according to Hoehn and Yahr (H&Y) were assessed by an experienced neurologist (H.O.). Patients did not discontinue medication, and the levodopa equivalent daily dose was on average 363 mg. They were clinically assessed at the Clinic for Cognitive Neurology at the University Hospital in Leipzig, Germany. Patients showed moderate symptoms of IPD, with an average H&Y stage of 2 (SD = 0.7) and a UPDRS of 37.7 (SD = 18.8). Inclusion criteria were low scores (<5; *M* = 1.29) on the Geriatric Depression Scale (Yesavage et al., 1982), absence of a hearing impairment, absence of musical training as assessed by a customized questionnaire for musical aptitudes. Additional neuropsychological testing included the Token-test (De Renzi and Vignolo, 1962), the consortium to establish a registry for Alzheimer’s disease (CERAD) (Welsh et al., 1994) and the Parkinson neuropsychometric dementia assessment (PANDA) (Kalbe et al., 2008). No other severe neurological or psychiatric illness was reported. Twenty (10 males) right-handed healthy adults, who matched the patients in age (*M* = 66.4, SD = 7.8) and education (*M* = 14.4 years; SD = 3.0) formed the control group. Healthy controls had no history of neurological or psychiatric disorders, showed no hearing impairments and did not actively practice music. All participants, recruited via the database of the Max Planck Institute for Human Cognitive and Brain Sciences, Leipzig, Germany, gave informed consent and were remunerated for their participation. The study was approved by the Ethics Committee of the University of Leipzig, Germany.

**Table 1:** **Demographic and clinical characteristics for IPD patients and healthy controls**.

	Patients		Controls	
	Mean (SD)	*n*	Mean (SD)	*n*
**Demographics**
Females	–	5	–	10
Males	–	10	–	10
**Handedness**
Right	–	15	–	20
Age	67.2 (7.5)	15	66.4 (7.8)	20
Years of education	14.7 (2.7)	15	14.4 (3.0)	20
Age at onset	59.3 (7.4)	15	–	–
Disease duration	7.9 (2.7)	15	–	–
**Clinical Characteristics**
UPDRS
I (mentation, behavior, and mood)	2.47 (1.8)	15	–	–
II (activities of daily living)	12 (6.11)	15	–	–
III (motor examination)	23.3 (12.4)	15	–	–
Total score	37.7 (18.8)	15	–	–
Hoehn and Yahr	2.0 (0.7)	15	–	–
0.5	–	1	–	–
1	–	2	–	–
2	–	6	–	–
2.5	–	5	–	–
3	–	1	–	–
Schwab and England	87.9 (5.8)	15	–	–
Medication
l-Dopa LED	136.0 (159.3)	14	–	–
Ago LED	253.0 (201.2)	14	–	–
Total LED	363.0 (260.5)	15	–	–

### Procedure

IPD patients took part in an auditory cuing training program. A 2-day assessment of perceptual and movement kinematics was administered before, after, and 1 month following the intervention. Controls were assessed only once. IPD patients were tested while they were in an ON-state. Details about the training program and the evaluation of perceptual and motor timing are provided in detail below.

### Training

The training sessions took place at the Day Clinic for Cognitive Neurology at the University of Leipzig. Patients were asked to walk while following a familiar German folk song. No explicit instructions to synchronize their footsteps to the beat of music were provided. The song was played without lyrics and the beat of the song was emphasized with a superimposed salient high-pitch bell sound. The beat rate of the auditory stimulus was set to ±10% of a patient’s spontaneous walking cadence as assessed prior to the first training session. The chosen beat rate [i.e., +10% (*n* = 8) or −10% (*n* = 7)] was the one which led to the longest step length as assessed in prior testing (Willems et al., 2006). Patients underwent three training sessions per week for 1 month. Medication was kept constant over the whole course of the study. Stimuli were delivered via a portable MP3-player (Sansa-Clip) and headphones (Sansa-Clip earbuds). Each training session lasted 30 min and consisted of three phases. In the first phase (10 min) the patient walked to the auditory rhythmic stimulus for 8 min. The stimulus was then stopped while the patient continued walking for 2 min at the same speed. In the second phase (10 min), the patient performed stop-and-go trials, in which the auditory stimulus was played for 30 s. At the end of the stimulus presentation, the patient stopped walking and restarted at the onset of the next stimulus presentation. During the last 2 min, the patient repeated the stop-and-go trials without the stimulus. The third phase (10 min) was the same as phase 1.

### Battery for the assessment of auditory sensorimotor and timing abilities (BAASTA)

Patients and controls were submitted to the BAASTA, which consists of a series of perceptual timing and motor timing tasks sketched out below. The test battery was administered over 2 days.

### Perceptual timing tasks

The first three tasks (duration discrimination, anisochrony detection with tones, and anisochrony detection with music) allow estimating thresholds of duration discrimination of two tones and to detect an interval embedded in an isochronous sequence of tones or in a musical excerpt. Thresholds are estimated using a maximum-likelihood adaptive procedure (MLP) (Green, 1993) implemented in the MLP toolbox (Grassi and Soranzo, 2009) in MATLAB. Participants performed 3 blocks of 16 trials each. In each trial, the stimulus difference was changed adaptively depending on the participants’ response. Thresholds corresponded to the midpoint of the psychometric curve defined as a probability of 63.1% of correct detection (Grassi and Soranzo, 2009). Stimuli were delivered via headphones (Sennheiser HD201) at a comfortable sound pressure level. A response was provided verbally by participants and entered by the experimenter via a computer keyboard. The tasks were preceded by four practice trials with feedback.

### Duration discrimination

The goal of this test is to measure the ability to discriminate two subsequent durations. The participants are presented with pairs of pure tones (frequency = 1 kHz; interval between tones = 600 ms). The first tone lasts 600 ms (standard duration) while the second tone (comparison) varies between 600 and 1000 ms. The duration of the second tone is controlled by the MLP algorithm. Participants’ task is to note if the second tone lasts “longer” than the first or has the “same” duration.

### Anisochrony detection with tones

This test assesses the sensitivity to time shifts (i.e., anisochrony) in a sequence of isochronous stimuli (Hyde and Peretz, 2003; Sowinski and Dalla Bella, 2013). Participants listen to sequences of five tones (tone frequency = 1047 Hz, duration = 150 ms). Isochronous sequences have a constant inter-onset-interval (IOI) while in non-isochronous sequences the fourth tone occurs earlier than expected based on the IOI of the preceding tones. This displacement results in reciprocal time shifts between tones 3/4 (shortened) and 4/5 (lengthened). The standard IOI is 600 ms. The magnitude of the local shift, up to 30% of the IOI (180 ms), is controlled by the MLP algorithm. After each sequence, participants are asked to judge whether the sequence was “regular” or “irregular.”

### Anisochrony detection with music

The purpose of the third task is to assess participants’ ability to detect a time shift (i.e., deviant beat) in a short musical excerpt (Sowinski and Dalla Bella, 2013). In each trial, a computer-generated musical excerpt is presented to participants. The excerpt is a two-bar fragment (i.e., eight quarter notes overall) taken from Bach’s “Badinerie” orchestral suite for flute BWV 1067, played with a piano timbre at a tempo of 100 beats/min (IOI = 600 ms; beat = quarter note). The IOI between musical beats is not manipulated in a regular sequence, while a local time shift (as in the previous task) is introduced at the onset of the fifth beat in an irregular sequence. The standard IOI between musical beats is 600 ms. The magnitude of the time shift, up to 30% (180 ms) of the IOI between musical beats, is controlled by the MLP algorithm. Participants’ task is to judge whether the sequence was “regular” or “irregular.”

### Beat Alignment Test

This task examines sensitivity to the beat conveyed by a musical stimulus. The task is an adapted version of the beat alignment task (Iversen and Patel, 2008; Fujii and Schlaug, 2013). Participants are presented with four musical excerpts including a salient beat structure. Two are fragments from Bach’s “Badinerie” and two from Rossini’s “William Tell Overture.” Each excerpt includes 20 beats (beat = quarter note). An isochronous sequence with a triangle timbre is superimposed on the music starting on the seventh beat. The isochronous sequence is either aligned to the musical beat or unaligned. In the latter case either relative phase is changed (with the tones preceding or following the beats by 33% of the IOI between beats, while keeping the same tempo of the musical stimulus), or period (with the tones being presented at a slower or faster rate by 10% of the quarter note duration). The 4 musical excerpts are presented at 3 different tempi (IOIs of 450, 600, and 750 ms, respectively), for a total of 24 beat-aligned trials and 48 beat-unaligned trials (72 trials overall). After each excerpt, participants are asked whether the isochronous sounds are aligned to the musical beat (perception of a regular pulse evoked by music).

### Motor timing tasks

Motor timing is assessed by hand tapping (Aschersleben, 2002; Repp, 2005). Participants are instructed to tap as regularly as possible with their right hand either without stimulation (unpaced tapping) or in the presence of a rhythmic auditory stimulus (paced tapping). Tapping is recorded via a Roland SPD-6 MIDI percussion pad controlled by MAX-MSP software (version 5.1). Stimuli are delivered over headphones (Sennheiser HD201) at a comfortable sound pressure level. No auditory feedback is provided during tapping. The tasks are preceded by practice trials.

### Unpaced tapping

The aim of this task is to assess the tapping rate and variability in the absence of a pacing stimulus. Participants are instructed to tap regularly at a comfortable rate for 60 s, while maintaining tapping rate as constant as possible. The same task is realized also with the left hand. Unpaced tapping tasks are repeated once more at the end of all the motor timing tasks of the BAASTA.

### Paced tapping to an isochronous sequence

This task assesses sensorimotor synchronization with isochronous sequences of tones. Participants are instructed to synchronize their taps to an isochronous sequence of 60 piano tones (tone frequency = 1319 Hz). The sequence is presented at three IOIs: 600, 450, and 750 ms. Each tapping trial at a given tempo is repeated twice.

### Paced tapping to music

In this task, the ability to synchronize to the beat of a musical stimulus is tested. Participants synchronize their taps to the beat of a well-formed musical excerpt from Bach’s “Badinerie” and from Rossini’s “William Tell Overture” (quarter note IOI = 600 ms), each including 64 beats. The tapping trial for each musical excerpt is repeated twice.

### Synchronization–continuation

The purpose of this test is to assess motor timing when participants continued tapping at a given rate after prior synchronization with an isochronous sequence (Wing and Kristofferson, 1973a,b; O’Boyle et al., 1996). Participants synchronize to a series of 10 piano tones presented isochronously at 3 tempi (600, 450, or 750 ms) and are instructed to continue tapping at the same rate (continuation phase) for a duration corresponding to 30 IOIs in the absence of a pacing stimulus. The end of the trial is indicated by a low-pitch tone. Each tapping trial at a given tempo is repeated twice.

### Adaptive tapping

This final test examines the ability to adapt to tempo change in a synchronization–continuation task, using an adaptive tapping task (Schwartze et al., 2011). Series of 10 tones are presented to participants. The first six tones of the sequences have an IOI of 600 ms, while the remaining four tones either maintain the same IOI or, in 67% of the trials, show a slower tempo (with a final IOI of 630 or 670 ms) or a faster tempo (with a final IOI of 570 or 525 ms). Participants are instructed to synchronize to the initial tempo, to adapt to the tempo change, and to continue tapping at the new tempo after the presentation of the last tone for a duration corresponding to 10 IOIs. At the end of each trial, participants are asked whether they perceived acceleration, deceleration, or no tempo change in the sequence. There are 10 blocks with 6 trials per block (4 with tempo change, 2 without), presented in random order.

### Gait assessment

Gait kinematics in the absence of auditory cues was assessed with a Vicon MX Motion Capture System during the second day of testing. Sixteen passive reflective markers (14 mm) were attached to participants’ lower body (four on the hip, three on each leg and foot, respectively) in accordance with the Conventional Gait Model (Baker, 2006). Participants were asked to walk for 1 min at their spontaneous walking speed. The trial was repeated twice. The performance was recorded using Vicon Nexus Software.

### Analysis

#### Perceptual timing tasks

In duration discrimination and anisochrony detection tasks, the smallest threshold value obtained across the three blocks (i.e., the best performance), expressed in percent of IOI (Weber Ratio) was retained as the final threshold. In the Beat Alignment Test (BAT), the number of Hits (i.e., when a misaligned metronome was correctly detected; maximum = 48 items) and of FAs (i.e., when a misalignment was erroneously reported; maximum = 24 items) was calculated. Trials with a FA rate higher than 30% were discarded. The percent of Hits minus FAs was computed to obtain an unbiased measure of detection performance.

#### Motor timing tasks

In the Unpaced tapping tasks, and in the continuation phase of the synchronization–continuation and adaptive tapping tasks accuracy of motor timing was obtained by computing the mean inter-tap interval (ITI). Tapping variability was calculated with the Coefficient of Variation (CV) of the ITI (i.e., the ratio of the SD of the ITIs over the mean ITI). In Paced tapping tasks, synchronization accuracy was obtained by calculating the mean absolute asynchrony (i.e., not signed) between the taps and pacing stimuli/beats. Small asynchrony indicates high accuracy. Synchronization variability is indicated by the standard error of asynchrony between taps and pacing stimuli. Both synchronization accuracy and precision are indicated in percent of the IOI. For both paced tapping and synchronization–continuation tasks, the results obtained in the trial showing the lowest variability were submitted to further analysis. Finally, in the adaptive tapping task, adaptation of tapping to the tempo change was measured with the adaptation index corresponding to the mean ITI of the continuation phase divided by the target ITI calculated for all tempi (see Schwartze et al., 2011). The adaptation indexes for faster (plus) and slower tempi (minus) were calculated separately. The sensitivity index (D-prime) for detecting tempo changes was also computed (Schwartze et al., 2011).

#### Statistical analysis

Since data were not normally distributed in both groups in more than 50% of the cases as assessed with Kolmogorov–Smirnov test, groups and condition were compared with non-parametric tests. To assess whether IPD patients were impaired prior to the training program, their performance was compared to that of controls with Mann–Whitney’s tests. If patients’ performance in the BAASTA was impaired at baseline, pre-/post-performance was compared with Wilcoxon matched-pairs tests. Patients’ individual performance was compared to that of controls via corrected *t*-tests (Crawford and Garthwaite, 2002).

## Results

### Gait

When walking at comfortable speed in the absence of an external cue, patients showed lower stride length (*M* = 980.4 mm) as compared to controls (*M* = 1152.3 mm) (*U* = 76, *p* < 0.01). Cueing training increased stride length (*M* = 1037.0 mm at post-test; *W*  = −70, *p* < 0.05) and this benefit was maintained 1 month after the training had ended (*M* = 1028.9 mm; *W*  = −78, *p* < 0.05).

### BAASTA

Before submitting data to the following analyses, trials were screened for outliers (e.g., for perceptual tasks, blocks with a higher false alarm rate higher than 30%; for motor tasks, taps with ITI deviating by more than three times the interquartile range from the median). A low number of outliers was found in both patients and controls. Overall in the perceptual tasks, 3.3% of the trials were rejected for patients and 3.8% for controls. In the motor tasks, <1% of taps were discarded for both patients and controls.

**Figure 1:**
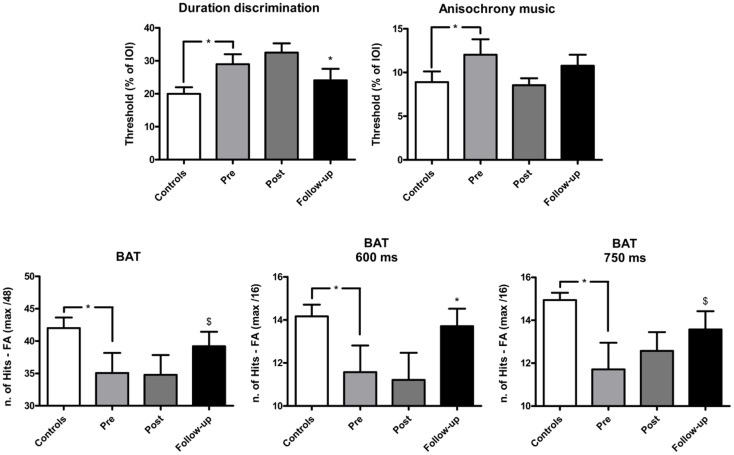
**Mean performance of IPD patients and controls in the perceptual tasks of the BAASTA**. Tasks where patients differed from controls before the cueing training are selectively reported. Error bars indicate the standard error of the mean (SEM). Note: *p < 0.05; ^$^marginally significant difference.

**Figure 2:**
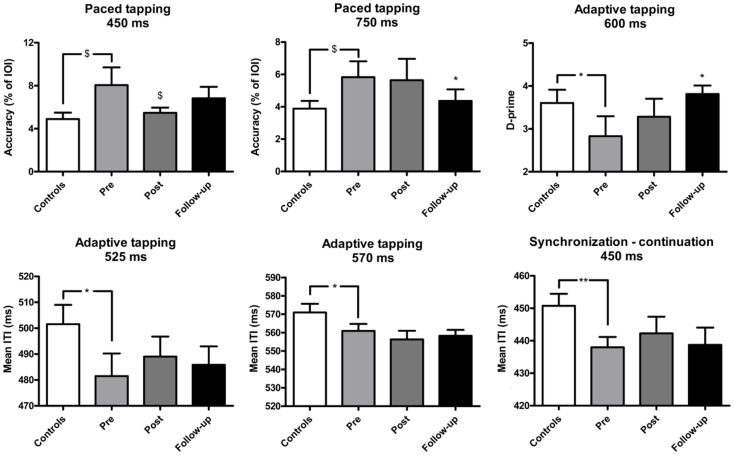
**Mean performance of IPD patients and controls in the motor tasks of the BAASTA**. Tasks where patients differed from controls before the cueing training are selectively reported. Error bars indicate the standard error of the mean (SEM). *p < 0.05; **p < 0.01; ^$^marginally significant difference.

The effect of training on perceptual and motor timing abilities was examined for the tasks of the BAASTA where patients showed impaired performance relative to controls pre-training. The mean results obtained in these tasks are shown in Figure 1 (perceptual tasks), and Figure 2 (motor tasks). Patients showed higher thresholds than controls (*U* = 75.5, *p* < 0.05) in the duration discrimination task before the training. Patients improved following the training, an effect that was not confirmed post-training but in the follow-up evaluation (*W*  = 66.0, *p* < 0.05). One month following the intervention, the difference between patients’ and controls’ discrimination thresholds was no longer significant. Patients displayed worse detection of anisochronies in musical stimuli than controls (*U* = 87.5, *p* < 0.05); yet, training did not improve patients’ performance in this task. Finally, in the BAT, patients had more difficulties in detecting misaligned beats before intervention when compared to controls (*U* = 74.5, *p* < 0.05). This difference was present for tempi with inter-beat-intervals of 600 and 750 ms (*U* = 84.5, *p* < 0.05 and *U* = 68.0, *p* < 0.05, respectively). No difference between patients (*M* = 11.8, SEM = 0.8) and controls (*M* = 12.9, SEM = 0.9) was found at the fastest tempo (450 ms). The detection of misaligned beats generally improved when tested at follow-up. The difference pre-/post-training just failed to reach significance (*W*  = −49.0, *p* = 0.07). This effect of training was mostly driven by patients’ performance at the average tempo (*W*  = −37.0, *p* < 0.05). Patients’ performance at the follow-up testing did not significantly differ from that of controls.

In the unpaced tapping tasks patients did not differ from controls before the training, in terms of accuracy (IPD: *M* = 580.4, SEM = 78.5; controls: *M* = 600.3, SEM = 63.9) and variability (IPD: *M* = 0.05, SEM = 0.08; controls: *M* = 0.05, SEM = 0.004). In paced tapping tasks, patients tended to be less accurate than controls when synchronizing with an isochronous sequence, a difference showing a statistical trend (at 450 ms, *U* = 102.0, *p* = 0.06; at 750 ms, *U* = 102.5, *p* = 0.06). The effect of the training was mostly visible in the follow-up session. Training led to increased synchronization accuracy with the isochronous sequences at the fastest tempo (450 ms) as confirmed by a statistical trend (*W*  = 50, *p* = 0.08). A significant increase of synchronization accuracy was found in the follow-up session (at 750 ms; *W*  = 72.0, *p* < 0.05). Patients’ performance in the follow-up evaluation did not statistically differ from that of controls. Patients and controls did not differ in terms of synchronization variability before the training (on average, IPD: *M* = 0.6, SEM = 0.1; controls: *M* = 0.6, SEM = 0.06). Patients and controls did not differ in terms of synchronization accuracy (on average, IPD: *M* = 7.3, SEM = 1.4; controls: *M* = 6.9, SEM = 0.8) and variability (on average, IPD: *M* = 0.8, SEM = 0.2; controls: *M* = 0.7, SEM = 0.08) when they synchronized with music.

In the synchronization–continuation task, patients tested prior to training were less accurate than controls only at the fastest tempo (450 ms, *U* = 78.0, *p* < 0.01). However, the two groups did not differ in terms of variability across all tempi (average variability, IPD: *M* = 0.03, SEM = 0.003; controls: *M* = 0.03, SEM = 0.002). Training had no effect on this task. Similar results were obtained in the adaptive tapping task. Before the training, patients exhibited lower accuracy than controls at the fastest tempi in the continuation phase (at 570 ms, *U* = 100.0, *p* < 0.05, and at 525 ms, *U* = 94.0, *p* < 0.05). Moreover, patients performed worse in detecting tempo changes at 600 ms (*U* = 99.5, *p* < 0.05). Both groups displayed similar tapping variability (on average, IPD: *M* = 0.05, SEM = 0.007; Controls: *M* = 0.05, SEM = 0.005), and comparable adaptation indexes (on average, IPD: *M* = 1.5, SEM = 0.2; Controls: *M* = 1.4, SEM = 0.1). Training was effective only in improving the detection of tempo changes, an effect visible when comparing pre-intervention to follow-up (*W*  = −43, *p* < 0.05). Patients’ perception in the follow-up session did not significantly differ from the performance of healthy controls.

Further analyses targeted the benefits of training on perceptual and motor timing at the individual level. Table 2 shows the individual performance of the 15 IPD patients on the BAASTA tasks showing significant effects at the group level. *z*-Scores for each testing session relative to the performance of controls are reported. Significant results are highlighted by the gray shading. Notably there are important individual differences in patients. After the training, some of them performed comparably to age-matched controls (*n* = 4), others showed improvement in perceptual/motor timing (*n* = 8) while the remaining did not respond to the training (*n* = 3). The percent of patients showing perceptual, motor, or perceptual and motor deficits at the three times of testing are summarized in Table 3. Perceptual or motor timing impairment is defined here based on the tasks included in Table 2. As can be seen, 73% of the patients displayed some form of timing impairment before the training. Post-intervention, poor timing abilities were found in 67% of the patients, while in the follow-up evaluation, only 40% of the patients still showed impaired timing.

**Table 2:** **Patients’ individual performances (*z*-scores) on the tasks of the BAASTA showing an effect of the cueing training at a group level**.

	Duration discrimination			BAT			D-prime 600 ms			Paced tapping accuracy			Paced tapping accuracy		
	*z*-Score			*z*-Score			*z*-Score			450 ms *z*-Score			750 ms *z*-Score		
Patient	Pre	Post	Follow-up	Pre	Post	Follow-up	Pre	Post	Follow-up	Pre	Post	Follow-up	Pre	Post	Follow-up
1	2.27	2.04	-0.14	-2.16	-2.16	-0.58	-0.23	0.43	0.43	-0.28	-0.40	2.28	-0.23	-0.48	-0.69
2	0.51	2.43	-0.47	-2.30	-2.02	-1.15	-1.11	-1.11	0.43	-0.46	0.26	-0.04	0.11	0.04	-0.42
3	0.44	-0.14	-0.14	0.72	0.86	0.86	0.43	0.43	0.43	0.43	0.30	0.33	-0.77	-0.90	-0.18
4	0.93	0.57	0.89	0.72	0.86	0.43	-0.75	-0.23	-0.23	-1.00	-0.37	0.18	-0.43	-0.61	-0.30
5	1.47	2.23	0.68	-1.87	-1.73	-0.43	0.42	0.43	0.43	1.22	0.26	-0.85	-0.50	-0.69	-0.97
6	-1.07	0.47	-0.53	0.00	-0.86	-1.30	-1.39	-0.23	0.43	4.56	1.88	3.54	-0.34	-0.46	-0.64
7	0.20	1.18	-0.75	NaN	NaN	NaN	0.43	-2.05	0.43	1.14	-0.53	0.36	2.30	0.59	0.40
8	2.27	3.36	1.62	-2.74	-2.16	0.14	0.43	0.43	-0.75	6.89	0.79	-0.51	3.86	0.30	0.38
9	-1.16	0.51	-0.59	0.29	0.43	0.14	-0.23	0.43	0.43	-0.34	-0.24	-1.05	-0.59	-0.26	-1.02
10	3.32	2.66	3.52	0.58	0.86	0.43	-1.11	-0.23	-0.10	1.12	0.71	2.27	1.93	8.74	3.25
11	0.23	2.59	4.30	-4.61	-3.74	-2.16	-3.78	0.49	0.43	4.69	0.12	3.19	5.12	2.39	2.45
12	3.48	-0.17	-0.56	0.58	0.00	0.72	0.43	0.43	0.43	-0.09	-0.80	-0.57	0.21	0.21	-0.74
13	1.07	3.23	-0.24	-0.86	-1.30	0.29	0.43	0.43	0.43	1.10	0.79	2.13	1.78	1.98	1.82
14	0.64	0.44	0.47	-2.30	-3.89	-3.31	-2.79	-3.58	-1.39	-0.42	0.58	-0.46	1.17	1.25	0.46
15	0.93	0.20	-1.00	0.58	0.29	0.29	0.43	0.43	0.43	-1.04	-0.11	-0.09	0.04	0.24	-0.46

*Values highlighted in gray indicate significant differences between the performances of patients and of healthy controls, as assessed with corrected t-tests (Crawford and Garthwaite, 2002). ‘NaN’ indicates missing values*.

*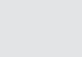 p < 0.05; 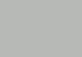 p < 0.01; 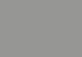 p < 0.001*.

**Table 3:** **Percentage of IPD patients who exhibited impaired perceptual and/or motor timing relative to healthy controls pre-, post-training, and at the follow-up**.

	Pre	Post	Follow-up
Perceptual only	5/15 (33%)	6/15 (40%)	1/15 (7%)
Motor only	3/15 (20%)	1/15 (7%)	3/15 (20%)
Perceptual and motor	3/15 (20%)	3/15 (20%)	2/15 (13%)
No impairment	4/15 (27%)	5/15 (33%)	9/15 (60%)

In summary, training successfully yielded improvements in gait kinematics, which were still present in follow-up tests 1 month after the training ended. Prior to training patients performed worse in several tests of the BAASTA. Training improved performance in three perceptual and two motor tasks at the group level. However, there were important individual differences: four patients were unimpaired at the training onset, eight patients improved their performance with training, while three did not respond to the intervention.

## Discussion

The main goal of the current study was to examine the effects of a 1-month auditory cueing gait-training program on perceptual and motor timing abilities in IPD patients. Performance was assessed with the BAASTA battery. Prior to the intervention, patients exhibited impaired perceptual timing across all BAASTA tasks except for anisochrony detection in isochronous sequences. On the contrary, motor timing was relatively spared, except lower accuracy in continuing tapping at a given rate, and in tapping along with an isochronous sequence. These findings are in line with previous evidence that IPD is associated with a malfunctioning timing system (Harrington et al., 1998, 2011; Spencer and Ivry, 2005; Smith et al., 2007; Koch et al., 2008; Merchant et al., 2008, 2013; Wearden et al., 2008; Jones and Jahanshahi, 2009). Moreover, we could confirm that auditory cueing has a beneficial effect on uncued gait by showing increased speed and step length. This effect outlasted the training (Marchese et al., 2000; Nieuwboer et al., 2001, 2007; Lehman et al., 2005). Our results show a stable effect of training on perceptual and motor timing tasks even after the training ended. In some tasks a delayed effect of cueing on perceptual and motor timing was observed (i.e., duration discrimination, BAT, paced tapping with a metronome, and adaptive tapping). The mechanisms leading to such delayed training effects require further study, for example, by controlling factors such as additional practice or placebo effects (for a discussion, see below).

Most notably, benefits of the training extended beyond gait, improving perceptual and motor timing abilities in a number of non-gait tasks assessed by the BAASTA. Benefits of auditory cueing on gait kinematics are likely to be mediated by a cerebello-thalamo-cortical network, which is also involved in timing (for reviews, see Kotz et al., 2009; Kotz and Schwartze, 2011). More specifically, projections from the SMA to the primary motor cortex may support motor output and modulate or stabilize gait kinematics over time. Compensation of a dysfunctional basal ganglia timing system via rhythmic auditory cues may be afforded by this compensatory cerebello-thalamo-cortical network (Sen et al., 2010). For example, evidence of hypermetabolism in the cerebellum of IPD patients, as a result of cueing training (del Olmo et al., 2006), provides preliminary support for this hypothesis. This circuitry plays a key role in domain-general timing (Kotz and Schwartze, 2011) and may underlie perceptual timing and coupling movement to an external pacing stimulus (Wing, 2002). Functional and/or structural changes in this compensatory network due to auditory cueing may affect both gait kinematics as well as perceptual and motor timing. Additional regions that may be associated with the observed timing benefits of cueing include temporal and parietal cortical areas (Nombela et al., 2013). For example, increased activation of the dentate nucleus near the midline and in the right temporo-parietal junction during a sensorimotor task was observed in IPD following auditory cueing training (del Olmo et al., 2006). The dentate nucleus has been linked to timekeeping (Malapani et al., 1998; Casini and Ivry, 1999) and the right inferior parietal and superior temporal cortex are involved in the coding of temporal intervals (Platel et al., 1997; Liegeois-Chauvel et al., 1998). Yet, the contribution of these regions to the benefits of auditory cueing is not clear to date and deserves further enquiry. In line with previous research, we find considerable variability between individual patients (Merchant et al., 2008). We observed a spectrum of individual profiles. Some patients were impaired in either or both perceptual and motor timing, while others performed comparable to healthy controls. For tasks positively affected by the training, eight patients showed an improvement after training, while six patients improved in perceptual timing, one in motor timing, and one in both timing functions. Four out of the six patients who improved in the BAT (beat-based timing) showed a comparable profile in the duration discrimination task (interval-based timing). Among the non-responders, 4 out of 15 showed no timing impairments prior to the training. Differences between patients may point to different loci of impairment within the neuronal network supporting perceptual and motor timing. The observed variability across tasks may be accounted for in the context of a hybrid model of timing, recently proposed by Merchant and collaborators (Merchant et al., 2008, 2013). The model postulates a partially distributed network, involving a core timing mechanism (e.g., cortico-thalamo-basal ganglia structures) and task-driven context-dependent mechanisms, engaged by specific behavioral contexts/tasks. A viable hypothesis is that performance variability between individuals and across tasks may therefore stem from the interaction between the core timing system and context- or task-dependent areas (for a discussion, see Merchant et al., 2013). A similar account may explain why in a functionally degenerated network, such as in IPD, training may show rather variable effects.

The study has some limitations that need to be addressed in further studies. One caveat is that the observed effects may be placebo effects in therapy. Indeed, placebo effects in PD can be very strong, and have been reported for dopamine release (e.g., de la Fuente-Fernandez et al., 2001). Moreover, there is a possibility that patients kept performing additional auditory cueing training at home after the end of the 1-month training session. We cannot exclude this possibility, even if patients were not encouraged to do so and even if the cueing device was not made available to the patients after the training. Finally, since BAASTA was administered three times for each patients, learning may act as a confound when considering the effects of training on perceptual and motor timing. Note, however, that the effects of the training on perceptual and motor timing abilities was selective, and confined to a subset of tasks of the BAASTA. Moreover, a thorough look at the individual performance of PD patients reveals different patterns of improvement due to training (e.g., delayed vs. immediate effects of training). These findings speak against a general explanation of improvements due to auditory cueing as a mere placebo, learning or practice effect. Indeed, these factors should indistinctly affect all tasks and patients in a similar fashion. Nevertheless, these factors should be carefully considered in further studies. A possibility which was not implemented in the present study is to include a control condition, where participants would perform a similar task in the absence of auditory cueing (e.g., music listening, or walking in the absence of a cue). This condition would allow pinpointing the contribution of coupling perception and action, which is characteristic of an auditory cueing training, compared to merely listening to music or uncued motor activity. In addition, submitting healthy participants to the BAASTA in a test–retest design will allow examining the contribution of practice and assessing which tasks of the battery are more susceptible to learning effects.

In summary, a training scheme relying on musically paced gait over 4 weeks in patients with mild to moderate IPD was shown to produce beneficial effects on perceptual and motor timing beyond gait. We suggest that such a generalization is mediated by a domain-general system, which governs perceptual and motor timing beyond gait. Such a network may be recruited when patients have to couple steps to auditory stimuli. These findings are relevant for theories about the functional and neuronal underpinnings of timing in performance and perception. However, they may also be considered as a first step toward the development of novel strategies for training cognitive aspects of IPD, extending beyond motor symptoms. Training targeted to cognitive functioning may be highly needed, since IPD has been increasingly recognized to not only affect movement but also cognition (Svenningsson et al., 2012). Training schemes bridging motor performance and cognition may be an important building block for devising efficient intervention strategies to delay cognitive decline in IPD.

## Conflict of Interest Statement

The authors declare that the research was conducted in the absence of any commercial or financial relationships that could be construed as a potential conflict of interest.

## References

[B1] Allman M. J. Meck W. H. (2012). Pathophysiological distortions in time perception and timed performance. Brain 135, 656–677 10.1093/brain/awr210 21921020 PMC3491636

[B2] Arias P. Cudeiro J. (2008). Effects of rhythmic sensory stimulation (auditory, visual) on gait in Parkinson’s disease patients. Exp. Brain Res. 186, 589–601 10.1007/s00221-007-1263-y 18214453

[B3] Aschersleben G. (2002). Temporal control of movements in sensorimotor synchronization. Brain Cogn. 48, 66–79 10.1006/brcg.2001.1304 11812033

[B4] Baker R. (2006). Gait analysis methods in rehabilitation. J. Neuroeng. Rehabil. 3, 4 10.1186/1743-0003-3-4 16512912 PMC1421413

[B5] Blin O. Ferrandez A. M. Serratrice G. (1990). Quantitative analysis of gait in Parkinson patients: increased variability of stride length. J. Neurol. Sci. 98, 91–97 10.1016/0022-510X(90)90184-O 2230833

[B6] Bloem B. R. (1992). Postural instability in Parkinson’s disease. Clin. Neurol. Neurosurg. 94(Suppl.), S41–S45 10.1016/0303-8467(92)90018-X 1320515

[B7] Bloem B. R. Hausdorff J. M. Visser J. E. Giladi N. (2004). Falls and freezing of gait in Parkinson’s disease: a review of two interconnected, episodic phenomena. Mov. Disord. 19, 871–884 10.1002/mds.20115 15300651

[B8] Casini L. Ivry R. B. (1999). Effects of divided attention on temporal processing in patients with lesions of the cerebellum or frontal lobe. Neuropsychology 13, 10–21 10.1037/0894-4105.13.1.10 10067771

[B9] Coull J. T. Cheng R. K. Meck W. H. (2011). Neuroanatomical and neurochemical substrates of timing. Neuropsychopharmacology 36, 3–25 10.1038/npp.2010.113 20668434 PMC3055517

[B10] Crawford J. R. Garthwaite P. H. (2002). Investigation of the single case in neuropsychology: confidence limits on the abnormality of test scores and test score differences. Neuropsychologia 40, 1196–1208 10.1016/S0028-3932(01)00224-X 11931923

[B11] de Bruin N. Doan J. B. Turnbull G. Suchowersky O. Bonfield S. Hu B. (2010). Walking with music is a safe and viable tool for gait training in Parkinson’s disease: the effect of a 13-week feasibility study on single and dual task walking. Parkinsons Dis. 2010, 483530 10.4061/2010/483530 20976086 PMC2957229

[B12] de la Fuente-Fernandez R. Ruth T. J. Sossi V. Schulzer M. Calne D. B. Stoessl A. J. (2001). Expectation and dopamine release: mechanism of the placebo effect in Parkinson’s disease. Science 293, 1164–1166 10.1126/science.1060937 11498597

[B13] De Renzi E. Vignolo L. A. (1962). The token test: a sensitive test to detect receptive disturbances in aphasics. Brain 85, 665–678 10.1093/brain/85.4.665 14026018

[B14] del Olmo M. F. Arias P. Furio M. C. Pozo M. A. Cudeiro J. (2006). Evaluation of the effect of training using auditory stimulation on rhythmic movement in Parkinsonian patients – a combine motor and [18F]-FDG PET study. Parkinsonism Relat. Disord. 12, 155–164 10.1016/j.parkreldis.2005.11.002 16459124

[B15] Elbaz A. Bower J. H. Maraganore D. M. Mcdonnell S. K. Peterson B. J. Ahlskog J. E. (2002). Risk tables for parkinsonism and Parkinson’s disease. J. Clin. Epidemiol. 55, 25–31 10.1016/S0895-4356(01)00425-5 11781119

[B16] Fujii S. Schlaug G. (2013). The Harvard Beat Assessment Test (H-BAT): a battery for assessing beat perception and production and their dissociation. Front. Hum. Neurosci. 7:771 10.3389/fnhum.2013.00771 24324421 PMC3840802

[B17] Grabli D. Karachi C. Welter M. L. Lau B. Hirsch E. C. Vidailhet M. (2012). Normal and pathological gait: what we learn from Parkinson’s disease. J. Neurol. Neurosurg. Psychiatr. 83, 979–985 10.1136/jnnp-2012-302263 22752693 PMC3852420

[B18] Grassi M. Soranzo A. (2009). MLP: a MATLAB toolbox for rapid and reliable auditory threshold estimation. Behav. Res. Methods 41, 20–28 10.3758/BRM.41.1.20 19182120

[B19] Green D. M. (1993). A maximum-likelihood method for estimating thresholds in a yes-no task. J. Acoust. Soc. Am. 93, 2096–2105 10.1121/1.406162 8473622

[B20] Hallett M. (2008). The intrinsic and extrinsic aspects of freezing of gait. Mov. Disord. 23(Suppl. 2), S439–S443 10.1002/mds.21836 18668625 PMC4758454

[B21] Harrington D. L. Castillo G. N. Greenberg P. A. Song D. D. Lessig S. Lee R. R. (2011). Neurobehavioral mechanisms of temporal processing deficits in Parkinson’s disease. PLoS ONE 6:e17461 10.1371/journal.pone.0017461 21364772 PMC3045463

[B22] Harrington D. L. Haaland K. Y. Hermanowicz N. (1998). Temporal processing in the basal ganglia. Neuropsychology 12, 3–12 10.1037/0894-4105.12.1.3 9460730

[B23] Howe T. E. Lovgreen B. Cody F. W. Ashton V. J. Oldham J. A. (2003). Auditory cues can modify the gait of persons with early-stage Parkinson’s disease: a method for enhancing parkinsonian walking performance? Clin. Rehabil. 17, 363–367 10.1191/0269215503cr621oa 12785243

[B24] Hyde K. L. Peretz I. (2003). “Out-of-pitch” but still “in-time”. An auditory psychophysical study in congenital amusic adults. Ann. N. Y. Acad. Sci. 999, 173–176 10.1196/annals.1284.023 14681135

[B25] Iversen J. R. Patel A. D. (2008). The Beat Alignment Test (BAT): surveying beat processing abilities in the general population. Proceedings of the 10th International Conference on Music Perception and Cognition; Sapporo, Japan 465–468

[B26] Jones C. R. Jahanshahi M. (2009). The substantia nigra, the basal ganglia, dopamine and temporal processing. J. Neural Transm. Suppl. 73, 161–171 10.1007/978-3-211-92660-4_13 20411776

[B27] Jones M. R. (1976). Time, our lost dimension: toward a new theory of perception, attention, and memory. Psychol. Rev. 83, 323–355 10.1037/0033-295X.83.5.323 794904

[B28] Kalbe E. Calabrese P. Kohn N. Hilker R. Riedel O. Wittchen H. U. (2008). Screening for cognitive deficits in Parkinson’s disease with the Parkinson neuropsychometric dementia assessment (PANDA) instrument. Parkinsonism Relat. Disord. 14, 93–101 10.1016/j.parkreldis.2007.06.008 17707678

[B29] Koch G. Costa A. Brusa L. Peppe A. Gatto I. Torriero S. (2008). Impaired reproduction of second but not millisecond time intervals in Parkinson’s disease. Neuropsychologia 46, 1305–1313 10.1016/j.neuropsychologia.2007.12.005 18215403

[B30] Koller W. C. Montgomery E. B. (1997). Issues in the early diagnosis of Parkinson’s disease. Neurology 49, S10–S25 10.1212/WNL.49.1_Suppl_1.S10 9222271

[B31] Kotz S. A. Schwartze M. (2011). Differential input of the supplementary motor area to a dedicated temporal processing network: functional and clinical implications. Front. Integr. Neurosci. 5:86 10.3389/fnint.2011.00086 22363269 PMC3277277

[B32] Kotz S. A. Schwartze M. Schmidt-Kassow M. (2009). Non-motor basal ganglia functions: a review and proposal for a model of sensory predictability in auditory language perception. Cortex 45, 982–990 10.1016/j.cortex.2009.02.010 19361785

[B33] Kwakkel G. De Goede C. J. Van Wegen E. E. (2007). Impact of physical therapy for Parkinson’s disease: a critical review of the literature. Parkinsonism Relat. Disord. 13(Suppl. 3), S478–S487 10.1016/S1353-8020(08)70053-1 18267287

[B34] Large E. W. (2008). “Resonating to musical rhythm: theory and experiment,” in The Psychology of Time, ed. Grondin S. (West Yorkshire: Emerald), 189–231

[B35] Large E. W. Jones M. R. (1999). The dynamics of attending: how we track time. Psychol. Rev. 106, 119–159 10.1016/j.neuroimage.2011.11.065 22155324

[B36] Lehman D. A. Toole T. Lofald D. Hirsch M. A. (2005). Training with verbal instructional cues results in near-term improvement of gait in people with Parkinson disease. J. Neurol. Phys. Ther. 29, 2–8 10.1097/01.NPT.0000282256.36208.cf 16386155

[B37] Lewis M. M. Slagle C. G. Smith A. B. Truong Y. Bai P. Mckeown M. J. (2007). Task specific influences of Parkinson’s disease on the striato-thalamo-cortical and cerebello-thalamo-cortical motor circuitries. Neuroscience 147, 224–235 10.1016/j.neuroscience.2007.04.006 17499933 PMC1939944

[B38] Liegeois-Chauvel C. Peretz I. Babai M. Laguitton V. Chauvel P. (1998). Contribution of different cortical areas in the temporal lobes to music processing. Brain 121(Pt 10), 1853–1867 10.1093/brain/121.10.1853 9798742

[B39] Lim I. Van Wegen E. De Goede C. Deutekom M. Nieuwboer A. Willems A. (2005). Effects of external rhythmical cueing on gait in patients with Parkinson’s disease: a systematic review. Clin. Rehabil. 19, 695–713 10.1191/0269215505cr906oa 16250189

[B40] Malapani C. Dubois B. Rancurel G. Gibbon J. (1998). Cerebellar dysfunctions of temporal processing in the seconds range in humans. Neuroreport 9, 3907–3912 10.1097/00001756-199812010-00026 9875727

[B41] Marchese R. Diverio M. Zucchi F. Lentino C. Abbruzzese G. (2000). The role of sensory cues in the rehabilitation of parkinsonian patients: a comparison of two physical therapy protocols. Mov. Disord. 15, 879–883 10.1002/1531-8257(200009)15:5<879::AID-MDS1018>3.0.CO;2-9 11009194

[B42] McIntosh G. C. Brown S. H. Rice R. R. Thaut M. H. (1997). Rhythmic auditory-motor facilitation of gait patterns in patients with Parkinson’s disease. J. Neurol. Neurosurg. Psychiatr. 62, 22–26 10.1136/jnnp.62.1.22 9010395 PMC486690

[B43] Merchant H. Harrington D. L. Meck W. H. (2013). Neural basis of the perception and estimation of time. Annu. Rev. Neurosci. 36, 313–336 10.1146/annurev-neuro-062012-170349 23725000

[B44] Merchant H. Luciana M. Hooper C. Majestic S. Tuite P. (2008). Interval timing and Parkinson’s disease: heterogeneity in temporal performance. Exp. Brain Res. 184, 233–248 10.1007/s00221-007-1097-7 17828600

[B45] Morris M. E. Huxham F. E. Mcginley J. Iansek R. (2001). Gait disorders and gait rehabilitation in Parkinson’s disease. Adv. Neurol. 87, 347–361 11347239

[B46] Nieuwboer A. De Weerdt W. Dom R. Truyen M. Janssens L. Kamsma Y. (2001). The effect of a home physiotherapy program for persons with Parkinson’s disease. J. Rehabil. Med. 33, 266–272 10.1080/165019701753236455 11766956

[B47] Nieuwboer A. Kwakkel G. Rochester L. Jones D. Van Wegen E. Willems A. M. (2007). Cueing training in the home improves gait-related mobility in Parkinson’s disease: the RESCUE trial. J. Neurol. Neurosurg. Psychiatr. 78, 134–140 10.1136/jnnp.200X.097923 17229744 PMC2077658

[B48] Nombela C. Hughes L. E. Owen A. M. Grahn J. A. (2013). Into the groove: can rhythm influence Parkinson’s disease? Neurosci. Biobehav. Rev. 37(10 Pt 2), 2564–2570 10.1016/j.neubiorev.2013.08.003 24012774

[B49] O’Boyle D. J. Freeman J. S. Cody F. W. (1996). The accuracy and precision of timing of self-paced, repetitive movements in subjects with Parkinson’s disease. Brain 119(Pt 1), 51–70 10.1093/brain/119.1.51 8624694

[B50] Platel H. Price C. Baron J. C. Wise R. Lambert J. Frackowiak R. S. (1997). The structural components of music perception. A functional anatomical study. Brain 120(Pt 2), 229–243 9117371 10.1093/brain/120.2.229

[B51] Repp B. H. (2005). Sensorimotor synchronization: a review of the tapping literature. Psychon. Bull. Rev. 12, 969–992 10.3758/BF03206433 16615317

[B52] Samii A. Nutt J. G. Ransom B. R. (2004). Parkinson’s disease. Lancet 363, 1783–1793 10.1016/S0140-6736(04)16305-8 15172778

[B53] Schwartze M. Keller P. E. Patel A. D. Kotz S. A. (2011). The impact of basal ganglia lesions on sensorimotor synchronization, spontaneous motor tempo, and the detection of tempo changes. Behav. Brain Res. 216, 685–691 10.1016/j.bbr.2010.09.015 20883725

[B54] Schwartze M. Kotz S. A. (2013). A dual-pathway neural architecture for specific temporal prediction. Neurosci. Biobehav. Rev. 37(10 Pt 2), 2587–2596 10.1016/j.neubiorev.2013.08.005 23994272

[B55] Sen S. Kawaguchi A. Truong Y. Lewis M. M. Huang X. (2010). Dynamic changes in cerebello-thalamo-cortical motor circuitry during progression of Parkinson’s disease. Neuroscience 166, 712–719 10.1016/j.neuroscience.2009.12.036 20034546 PMC2852615

[B56] Sharma A. Szeto K. Desilets A. R. (2012). Efficacy and safety of deep brain stimulation as an adjunct to pharmacotherapy for the treatment of Parkinson disease. Ann. Pharmacother. 46, 248–254 10.1345/aph.1Q508 22234991

[B57] Smith J. G. Harper D. N. Gittings D. Abernethy D. (2007). The effect of Parkinson’s disease on time estimation as a function of stimulus duration range and modality. Brain Cogn. 64, 130–143 10.1016/j.bandc.2007.01.005 17343966

[B58] Sowinski J. Dalla Bella S. (2013). Poor synchronization to the beat may result from deficient auditory-motor mapping. Neuropsychologia 51, 1952–1963 10.1016/j.neuropsychologia.2013.06.027 23838002

[B59] Spaulding S. J. Barber B. Colby M. Cormack B. Mick T. Jenkins M. E. (2012). Cueing and gait improvement among people with Parkinson’s disease: a meta-analysis. Arch. Phys. Med. Rehabil. 94, 562–570 10.1016/j.apmr.2012.10.026 23127307

[B60] Spencer R. M. Ivry R. B. (2005). Comparison of patients with Parkinson’s disease or cerebellar lesions in the production of periodic movements involving event-based or emergent timing. Brain Cogn. 58, 84–93 10.1016/j.bandc.2004.09.010 15878729

[B61] Svenningsson P. Westman E. Ballard C. Aarsland D. (2012). Cognitive impairment in patients with Parkinson’s disease: diagnosis, biomarkers, and treatment. Lancet Neurol. 11, 697–707 10.1016/S1474-4422(12)70152-7 22814541

[B62] Thaut M. H. McIntosh G. C. Rice R. R. Miller R. A. Rathbun J. Brault J. M. (1996). Rhythmic auditory stimulation in gait training for Parkinson’s disease patients. Mov. Disord. 11, 193–200 10.1002/mds.870110213 8684391

[B63] Thaut M. H. McIntosh K. W. McIntosh G. C. Hoemberg V. (2001). Auditory rhythmicity enhances movement and speech motor control in patients with Parkinson’s disease. Funct. Neurol. 16, 163–172 11495422

[B64] Wearden J. H. Smith-Spark J. H. Cousins R. Edelstyn N. M. Cody F. W. O’Boyle D. J. (2008). Stimulus timing by people with Parkinson’s disease. Brain Cogn. 67, 264–279 10.1016/j.bandc.2008.01.010 18329150

[B65] Welsh K. A. Butters N. Mohs R. C. Beekly D. Edland S. Fillenbaum G. (1994). The consortium to establish a registry for Alzheimer’s disease (CERAD). Part V. A normative study of the neuropsychological battery. Neurology 44, 609–614 8164812 10.1212/wnl.44.4.609

[B66] Willems A. M. Nieuwboer A. Chavret F. Desloovere K. Dom R. Rochester L. (2006). The use of rhythmic auditory cues to influence gait in patients with Parkinson’s disease, the differential effect for freezers and non-freezers, an explorative study. Disabil. Rehabil. 28, 721–728 10.1080/09638280500386569 16809215

[B67] Wing A. M. (2002). Voluntary timing and brain function: an information processing approach. Brain Cogn. 48, 7–30 10.1006/brcg.2001.1301 11812030

[B68] Wing A. M. Kristofferson A. B. (1973a). Response delays and the timing of discrete motor responses. Percept. Psychophys. 14, 5–12 10.3758/BF0319860711543514

[B69] Wing A. M. Kristofferson A. B. (1973b). The timing of interresponse intervals. Percept. Psychophys. 13, 455–460 10.3758/BF03205802

[B70] Wu T. Wang J. Wang C. Hallett M. Zang Y. Wu X. (2012). Basal ganglia circuits changes in Parkinson’s disease patients. Neurosci. Lett. 524, 55–59 10.1016/j.neulet.2012.07.012 22813979 PMC4163196

[B71] Yesavage J. A. Brink T. L. Rose T. L. Lum O. Huang V. Adey M. (1982). Development and validation of a geriatric depression screening scale: a preliminary report. J. Psychiatr. Res. 17, 37–49 10.1016/0022-3956(82)90033-4 7183759

